# Assessment of Heavy Metal Contamination and Associated Health Risks in Honey from Kellem Wollega Zone, Ethiopia

**DOI:** 10.3390/toxics14030229

**Published:** 2026-03-08

**Authors:** Aschalew Nega Teferi, Yibrehu Bogale Dibabe, Abbay Gebretsadik Debalke, Teshager Worku Beyene, Weiying Feng, Chiamin Ho

**Affiliations:** 1 School of Material Science and Engineering, Beihang University, Beijing 100191, China; aschalewnega@buaa.edu.cn (A.N.T.);; 2 Department of Chemistry, Haramaya University, Dire Dawa 138, Ethiopia; 3 Department of Applied Chemistry, Adama Science and Technology University, Adama 1888, Ethiopia; 4 Department of Biology, Bahir Dar University, Bahir Dar 79, Ethiopia; teshagerworku2008@gmail.com

**Keywords:** carcinogenic risk, environmental contamination, heavy metals, honey, non-carcinogenic risk

## Abstract

Honey is consumed worldwide for its nutritional and medicinal value, but it can also expose people to toxic metals from environmental contamination. This study analyzes heavy metal levels and assesses health risks using inductively coupled plasma optical emission spectrometry (ICP-OES) in honey collected from three areas in the Kellem Wollega Zone, Ethiopia: Dambi Dollo, Gawo Kebe, and Anafilo. The concentrations followed the order: Zn > Fe > Pb > Mn > Cu > Ni > Cd. Notably, Pb and Cd levels exceeded the WHO/FAO maximum permissible limits. The assessment of non-carcinogenic health risks for adult consumers based on the average daily dose, target hazard quotient, and hazard index indicated that all calculated values were below the critical threshold of 1. This result suggests that honey consumption poses no significant non-carcinogenic risk. In contrast, the estimated target cancer risk and cumulative cancer risk (∑TCR) exceeded safety thresholds, indicating potential moderate lifetime carcinogenic risk from chronic exposure. Likely sources of high metal levels include local mining activities, agricultural inputs, and improper honey storage. Consequently, these findings highlight the need for continuous environmental monitoring, stricter regulations, and improved apicultural practices to ensure honey safety and protect public health.

## 1. Introduction

Honey is a natural, sweet, and viscous fluid produced by Apis mellifera bees from nectar plants [1,2]. A nutritious food of great economic importance worldwide [3], honey is a complex mixture of major and minor nutrients, including significant amounts of proteins, carbohydrates, flavonoids, minerals, waxes, enzymes, and vitamins [4,5,6]. Honey has antioxidant, antibacterial, immunity-enhancing, and anti-inflammatory properties, as well as other physiological activities [7,8,9]. Foraging honeybees cover about 7 km^2^ and encounter pollutants, including pesticides, heavy metals, and radioactive substances, from contaminated plants, soil, water, air, and flora [10,11]. Consequently, honey produced by these bees is a complex mixture comprising plant-derived nutrients and secondary compounds, as well as both organic and inorganic environmental impurities [12]. Heavy metals in the environment come from natural sources (forest fires, sea spray, soil dust) and anthropogenic activities (industrial emissions, agrochemical use, traffic, waste discharge) [13,14]. Notable sources are agrochemicals such as organic mercury, cadmium-based fertilizers, and arsenic-containing pesticides [15]. Contamination may also occur during honey harvesting, processing, or storage, further emphasizing the need for systematic monitoring of honey safety [9]. Elemental contaminants of honey also come from flowers in botanical gardens with naturally or artificially contaminated geographical origins [16]. Essential elements (Fe, Mn, Cu, Zn), among others, play vital roles in metabolism and health when ingested at levels not exceeding their recommended dietary intake; however, at levels above tolerance levels, they become hazardous to human health. In contrast, non-essential metals like Pb and Cd are particularly hazardous, being associated with carcinogenic, neurotoxic, and organ-damaging effects [17,18].Heavy metal accumulation in honey poses a potential risk to food safety and consumer health. Elements like Pb and Cd cannot be metabolized; they accumulate in the body over time, leading to chronic toxicity and increased cancer risk even at low concentrations [19]. Honey serves as a sensitive bioindicator of ecosystem pollution, reflecting environmental contamination levels in the region where bees forage [20]. These approaches more effectively identify potential health hazards and support accurate exposure evaluation and regulatory decisions [21].

In Ethiopia, apiculture is a vital economic activity, ranking it among the top ten honey producers in the world and a major producer on the African continent [22,23]. The Kellem Wollega Zone in western Ethiopia is a key honey-producing area due to its favorable climate and widespread apiculture. Honey is widely consumed for its nutritional and therapeutic properties. It is an important part of the local diet. However, environmental pollution can cause both essential and non-essential heavy metals to accumulate in honey, posing health risks. However, no study has comprehensively assessed heavy metal contamination and related health risks in honey from this area. This highlights the need for regular monitoring to improve food safety and guide evidence-based practices. This study provides the first baseline dataset on heavy metal contamination in honey from the Kellem Wollega Zone and demonstrates its potential as an environmental biomonitoring tool. Although regionally focused, the findings are internationally relevant, as honey is globally consumed and traded, and metal contamination poses shared environmental and food safety challenges. The objectives of this study were to: (1) quantify the levels of selected heavy metals (Cu, Fe, Mn, Ni, Zn, Cd, and Pb) in honey collected from three districts of the Kellem Wollega Zone using ICP-OES and (2) evaluate the associated non-carcinogenic and carcinogenic health risks for adult consumers. These findings provide critical baseline data to inform evidence-based environmental monitoring and food safety actions, guide policymakers, and strengthen comparative research on honey contamination worldwide.

## 2. Materials and Methods

### 2.1. Study Area

**Figure 1:**
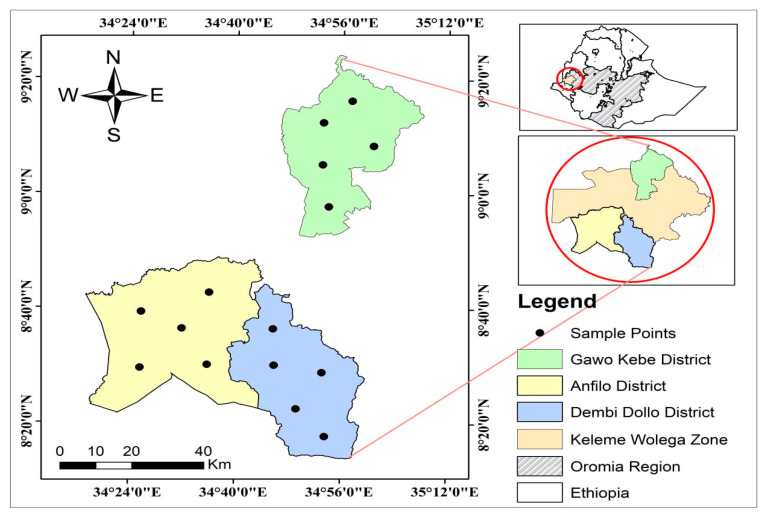
Map of study area.

This investigation was conducted in three select districts of the Kellem Wollega Zone in Ethiopia: Dambi Dollo, Gawo Kebe, and Anfillo. Dambi Dollo is located approximately 652 km west of Addis Ababa. The town has an elevation of 1701–1827 m above sea level and a hot, humid tropical climate, with an average annual rainfall of 700–1100 mm [24]. Gawo Kebe is bordered by the West Wollega Zone to the east and north, Jimma Horo to the west, Yemalogi Welele to the south, and Dale Wabera to the southeast. Anfillo is found in the southwestern part of the zone, adjacent to the Gambela Region in the southwest, Jimma Gidami to the north, Yemalogi Welele to the northeast, and Sayo to the east [25], see Figure 1.

### 2.2. Sample Collection and Preparation

Honey samples were collected from three districts (woredas) of the Kellem Wollega Zone, Oromia, Ethiopia. A total of fifteen initial subsamples were obtained. Using a cluster sampling design to capture geographic variability, five apiary points were selected within each district, and one subsample was collected from each point. The five subsamples from each district were then composited into a single representative sample, yielding a total of three composite samples for laboratory analysis. Each composite sample was stored in a clean, labeled glass jar, refrigerated at Dambi Dollo University, and subsequently transported under cold chain conditions to Bahir Dar University for storage at 4 °C until analysis. Crystallized samples were gently liquefied in a 4 °C water bath prior to digestion to ensure homogeneity and preserve heat-sensitive components.

### 2.3. Reagents and Chemicals

All chemicals and reagents were high-purity, analytical- grade. Concentrated HNO_3_ (69–72%) and HClO_4_ (70%) from UNI-CHEM^®^ (Mumbai, India) were used for sample digestion. Calibration used a certified multi-element standard (Cu, Fe, Mn, Ni, Zn, Cd, Pb, 1000 mg L^−1^ in 2% HNO_3_, Buck Scientific PuroGraphic, Norwalk, CT, USA). Solutions were prepared with deionized water (≤1.5 μS cm^−1^).

### 2.4. Instruments and Apparatus

The laboratory devices that were used for this study include: An analytical balance (Denver Instrument Company Model AA-200DS, ±0.0001 g), Digestion furnace (Model KDN-20C, Hinotek, Ningbo, China), Kjeldahl tubes fitted with a reflux condenser were used in the Kjeldahl digestion block apparatus, hot plate, water bath, volumetric flasks, beakers, measuring cylinders, A refrigerator (Beko RDP 6900, Kawasaki, Kanagawa, Japan), spatula, funnel, filter papers, pipettes and micropipettes, round bottom flask, A 0.45 µm type membrane filter paper (Whatman^®^, No. 41, Maidstone, England, UK) and Inductively Coupled Plasma Optical Emission Spectrometer (ICP-OES, PerkinElmer Optima 8000, New York, NY, USA) was used heavy metal analysis.

### 2.5. Analytical Procedure

#### 2.5.1. Sample Digestion for Multi-Element Analysis Using ICP-OES

0.5 g of honey samples were weighed using a digital analytical balance and transferred into a 100 mL round-bottom digestion flask. A total of 3.5 mL of acid mixture (2 mL HNO_3_ (65–68%) and 1.5 mL HClO_4_ (70%), UNI-CHEM^®^, Mumbai, India), 2:1.5 ratio was added. The mixture was swirled gently and then fitted with a reflux condenser. Digestion was conducted on a Kjeldahl block at 160–240 °C, with 240 °C as the optimum temperature, yielding a clear solution after 2 h 45 min. Conditions such as clarity, minimal reagent use, and efficient time and temperature were selected for completeness [26,27]. After digestion, the solution was cooled for 10 min with, then 10 min without, the condenser. Deionized water was added to dissolve any precipitate. The digest was filtered through Whatman No. 41 paper and transferred to a 100 mL volumetric flask. The digestion flask was rinsed various times with deionized water, and the rinse solutions were combined. The solution was then diluted at volume with deionized water and stored at 4 °C until ICP-OES analysis. Each sample was digested in triplicate along with a reagent blank.

#### 2.5.2. Preparation of Stock and Working Standard Solutions

Standard solutions of each metal (Cu, Fe, Mn, Ni, Zn, Cd, and Pb) were prepared at a concentration of 1000 mg/L. To prepare intermediate metal standard solutions (100 mg/L each), 10 mL of the 1000 mg/L standard solution of each metal was diluted with distilled water (boiled for 30 min) to a final volume of 100 mL [28].

#### 2.5.3. Analysis of Heavy Metals by ICP-OES

Levels of seven heavy metals (Cu, Fe, Mn, Ni, Zn, Cd, and Pb) in digested honey samples were analyzed using ICP-OES. To ensure accuracy, the device was first calibrated with a blank and matrix-matched standards. Following calibration, each digested sample was diluted 1:10 with deionized water before measurement. All samples and blanks were then measured in triplicate. For quantification, each metal was analyzed using a calibration curve based on emission intensity, and concentrations were calculated using the regression equation. Finally, Table 1 summarizes the ICP-OES operating parameters.

### 2.6. Analytical Method Performance Validation

To validate the analytical method’s performance for quantifying metals, the instrumental detection limit (IDL) was determined as three times the standard deviation of the reagent blank (IDL = 3 × SD_blank_). The method detection limit (MDL) was calculated by multiplying the standard deviation from replicate sample measurements by the student’s t-value (MDL = SD × T-test value), ensuring 99% confidence throughout the method [29]. Also, the limit of quantification (LOQ) was set at 10 times the standard deviation of the blank (LOQ = $10\;\times \;{\mathrm{SD}}_{\mathrm{blank}}$), defining the lowest concentration measurable with acceptable accuracy and precision [30]. Furthermore, the linear and working ranges, accuracy, precision, and recovery study of spiked standards were used. The calibration standards were prepared in six concentrations (0.05, 1.05, 2.05, 3.05, 4.05, and 5.05 mg/L). The coefficient of determination (*R*^2^) ranged from 0.995 to 0.999, indicating a linear relationship between absorbance and concentration [31]. The IDL, MDL, and LOQ values ranged from 0.014–2.144 mg/L, 0.028–2.655 mg/L, and 0.057–7.157 mg/L, respectively (Table 2). The consistent IDL < MDL < LOQ order and low MDL values demonstrate the method’s robustness and support reliable quantification [32].

Method accuracy was validated through spike-recovery analysis, adding known concentrations of target analytes to the samples prior to digestion [32]. The recovery percentage was calculated as

(1) $$\%\mathrm{Recovery}=\frac{\;C_{spiked\;sample}-\;\;C_{unspiked\;sample}}{Actual\;spike\;Concentration\;}\;\times \;100$$

Method precision was assessed as the repeatability of measurements on a homogeneous sample, expressed as the relative standard deviation (*RSD*)

(2) $$\%\mathrm{RSD}=\frac{\mathrm{Standard}\;\mathrm{deviation}}{\mathrm{Mean}\;\mathrm{value}}\times 100$$

Heavy metal recoveries were measured by adding standard solutions of Cu, Fe, Mn, Ni, Cd, Zn, and Pb to the samples. The method demonstrated high accuracy, with recovery rates for all metals ranging from 89.1% to 114.8%, which is within the accepted 80–120% range for metal analysis in (Table 3). Excellent precision was also confirmed, with relative standard deviation (%RSD) values for replicates ranging from 0.12% to 1.35%; all were less than 20%, indicating the applicability of the suggested approach [33]. These recovery rates indicate strong agreement between the measured and expected values for most metals.

### 2.7. Methods of Health Risks Assessment

#### 2.7.1. Methods of Non-Carcinogenic Health Risk

The potential non-carcinogenic health risk to honey consumers was evaluated using the United States Environmental Protection Agency (USEPA) risk assessment model [34,35]. The average daily dose (ADD, mg/kg/day) of metals ingested through honey consumption was calculated [36].

(3) $$\mathrm{ADD}=\frac{Ci\times IR\times EF\times ED}{BW\times AT}$$

where *Ci* is the concentration of metals estimated in the honey sample (mg/kg), *IR* is the ingestion rate of honey (5.5 g/day) [37], *EF* is the exposure frequency (365 days/year) [38], *ED* is the exposure period (70 years), *BW* is the average body weight of an adult of 67 kg [19], and *AT* is the average exposure period (*ED* × 365 days/year) [39].

The target hazard quotient (THQ) is calculated to evaluate the potential non-carcinogenic risk associated with heavy metal exposure, using the oral reference dose (*RfD*, mg/kg/day) as a reference value [40]. The target hazard quotient (THQ) was calculated using the methods described in [41].

(4) $$\mathrm{THQ}=\;\frac{ADD}{RfD}$$

where the oral reference dose (*RfD*) was used as the toxicity benchmark. Metal-specific *RfD* values were selected through a tiered approach to maintain regulatory consistency and validity. For metals with values available in the USEPA Integrated Risk Information System (IRIS), the following *RfD* were used: Mn (0.14), Ni (0.02), Cd (0.001), and Zn (0.3) [42,43,44,45]. For metals not listed in IRIS, *RfD* were sourced from authoritative regulatory documents or peer-reviewed literature, including Cu (0.04) from the U.S. EPA HEAST/Superfund ROD table [46] and Fe (0.7) and Pb (0.0035) from peer-reviewed studies [47,48] respectively.

The non-carcinogenic risk from combined metal exposure was assessed using the hazard index (HI), calculated as the sum of the *THQs* for all metals [41].

(5) $$\mathrm{HI}={THQ}_{Cu}+{THQ}_{Fe}+{THQ}_{Mn}+{THQ}_{Ni}+{THQ}_{Zn}+{THQ}_{Cd}+{THQ}_{Pb}$$

#### 2.7.2. Methods of Carcinogenic Health Risk

Carcinogen risks were estimated as the cumulative chance that a person would get cancer if exposed to that possible carcinogen over their lifetime [38]. Carcinogens have allowable levels of risk ranging from $1.0\times 10^{-4}\;to\;1.0\times 10^{-6}.$ The target cancer risk (TCR) for each metal was calculated using Equation (6):

TCR = ADD × CSF (6)

where CSF is the cancer slope factor for toxic heavy metals. The oral CSF for Cd, Ni, and Pb was 0.38, 1.7, and 0.0085 [41,49].

### 2.8. Statistical Analysis

Statistical analyses were performed using IBM SPSS Statistics software (Version 26.0) [50]. One-way analysis of variance (ANOVA), followed by Tukey’s HSD post hoc test, was used to determine significant differences in heavy metal concentrations among honey sampling sites. A significance level of *p* < 0.05 was applied for all analyses.

## 3. Results and Discussion

### 3.1. Levels of Heavy Metals in Honey Samples

Heavy metal concentrations in honey from Dambi Dollo, Gawo Kebe, and Anafilo are shown as mean ± standard deviation in Table 4. Statistically significant differences among sites were observed for Cu, Fe, Mn, Ni, Zn, and Cd (*p* < 0.05), while Pb levels showed no significant difference (*p* > 0.05), as analyzed by one-way ANOVA.

Cu is essential for energy production, nerve conduction, and the health of connective tissues, the immune system, and the neurological system [51]. The mean Cu concentration ranged from 6.361 to 12.788 mg/kg. Tukey’s HSD post hoc test revealed statistically significant differences in Cu levels between Dambi Dollo and Gawo Kebe, Dambi Dollo and Anafilo, and Gawo Kebe and Anafilo (*p* < 0.05). This study found that the concentrations of the target metals in the honey samples were below the average daily intake of 30 mg from food sources, which is the recommended amount [51]. However, the Cu concentration observed in all samples exceeded the Codex recommendation limit of 5 µg/g [52]. The Cu content analyzed in present study was comparatively lower than values reported for Turkish honeys, which ranged from (0.22–198.38 mg/kg) [53], but higher than concentrations documented in honeys from Switzerland (0.051 to 3.317 mg/kg) [6], Romania (0.064 to 0.549 mg/kg) [54], Armenia (0.09 to1.369 mg/kg) [55], and Australian (0.05 to 4.8 mg/kg) [56]. The increased Cu concentration in the study locations may be ascribed to multiple anthropogenic activities.

The Fe concentrations ranged from 99.575 to 159.670 mg/kg. Tukey’s HSD post hoc test revealed statistically significant differences in Fe concentrations among the following honey sample pairwise comparisons: Dambi Dollo with Gawo Kebe, Dambi Dollo with Anafilo, and Gawo Kebe with Anafilo (*p* < 0.05) in (Table 4). The analysis indicated that Fe concentrations in all sampled honey significantly exceeded the 15 mg/kg maximum limit established by the Codex Alimentarius [52]. International health guidelines established by the FAO and WHO indicate that an individual weighing 60 kg should have a preliminary tolerated daily intake of 48 mg of Fe [57]. The Fe levels in this study are lower than those reported for Turkish honeys, which ranged from (3.506 to 1278.779 mg/kg) [53]. However, the obtained Fe levels were higher than those documented in honeys from the Tigray Region of northern Ethiopia (5.32–28.6 mg/kg) [58]. The results show that the levels of Mn in honey samples ranged from 10.234 to 16.725 mg/kg.

The results show that Mn levels in honey samples ranged from 10.234 to 16.725 mg/kg. The Mn levels in Gawo Kebe and Dembi Dollo Market honey were comparable, while the Anafilo sample had the lowest concentration. Based on Tukey’s HSD post hoc test, the samples revealed no statistically significant difference in Mn concentrations between Dambi Dollo and Gawo Kebe (*p* > 0.05). However, significant differences were observed between Dambi Dollo and Anafilo and between Gawo Kebe and Anafilo (*p* < 0.05), as shown in (Table 4). Our results are higher than those reported by [59],which ranged from 0.001 to 0.274 mg/kg. Similarly, in the present study, the measured Mn concentration was higher than that reported by [60],which ranged from 0.11 to 7.22 mg/kg. The Mn levels in this study fall within the broader range 0.096–29.496 mg/kg reported for Turkish honeys [53]. Therefore, the elevated metal concentrations in this study can be attributed to either the honey production process or various anthropogenic activities in the surrounding areas.

The results show that the Ni levels in honey samples ranged from 6.047 to 8.853 mg/kg. Based on Turkey’s HSD post hoc test, the samples revealed no statistically significant difference in Ni concentrations between Dambi Dollo and Gawo Kebe (*p* > 0.05). However, significant differences were observed between Dambi Dollo and Anafilo, and between Gawo Kebe and Anafilo (*p* < 0.05), as shown in Table 4. The Ni concentration in our results was higher than the range reported for Pakistan (0.06–0.33 mg/kg) [61], Iran (0.065–1.094 mg/kg) [62], and Romania (0.101–0.570 mg/kg) [54]. Although Ni is widespread, its level in honey varies by region. Soil bioavailability affects how nectar-producing plants absorb Ni, and air deposition influences the surfaces of blossoms. The results show that Zn levels in honey samples ranged from 75.713 to 209.543 mg/kg. Based on Turkey’s HSD post hoc test, statistically significant differences were observed between the Zn concentrations in Dambi Dollo and Gawo Kebe, Dambi Dollo and Anafilo, as well as between Gawo Kebe and Anafilo (*p* < 0.05), as shown in Table 4. Our results align with the range of 1.73–245.21 mg/kg reported for Turkey honey by Altunatmaz et al. [53]. However, the zinc concentrations observed in this study were significantly higher than those reported for Australian honeys (0.16–120 mg/kg) [56]. The elevated Zn concentrations in honey may be associated with agricultural activities, particularly the use of Zn-containing soil amendments to address nutrient deficiencies and boost crop production, which suggests a likely anthropogenic impact on the local geochemical cycle [63].

Cd concentrations in honey samples ranged from 1.38 to 2.30 mg/kg. Based on Turkey’s HSD post hoc test, statistically significant differences were observed between the Cd levels in Dambi Dollo and Gawo Kebe, Dambi Dollo and Anafilo, as well as between Gawo Kebe and Anafilo (*p* < 0.05), as shown in Table 4. The significant difference in Cd concentrations among the sampling sites (*p* < 0.05) indicates that geographical location is a major determinant of Cd content in honey. All measured levels far exceed the maximum permissible limit of 0.05 mg/kg for Cd in honey, as set by the European Commission (Regulation (EU) 2023/915) [64]. Furthermore, for a 60 kg adult, the estimated intake corresponds to a level that exceeds the provisional tolerable monthly intake (PTMI) of 25 µg/kg body weight established by the Codex Alimentarius and the Joint FAO/WHO Expert Committee on Food Additives [52,65]. The Cd concentrations in this study are higher than those reported for honey from other studies: Romanian (0.001–0.012 mg/kg) [54],Turkey (0.000–0.300 mg/kg) [53], and the Oromia Special Zone in Ethiopia (0.04–0.70 mg/kg) [66]. The elevated Cd levels observed in these areas are likely attributable to anthropogenic activities, such as the application of phosphate fertilizers and the use of cadmium-based pigments and plastics, which can leach into the environment or directly into products like honey from packaging [56]. Based on our findings, Pb concentration ranged from 1.65 to 21.421 mg/kg, as shown in Table 4. There was no significant difference (*p* > 0.05) between Pb levels across each area. The Pb levels in all samples substantially exceeded the Codex Alimentarius maximum limit of 0.1 mg/kg [62]. Moreover, the concentrations measured in this study were also higher than the reported ranges for honeys from the Oromia Special Zone, Ethiopia (0.37–0.90 mg/kg) [66] and Armenia (0.002–0.045 mg/kg) [55]. The elevated Pb levels in these areas, particularly the peak concentration in Anafilo, can be attributed to the region’s intensive gold mining industry, a known source of Pb contamination through mines and waste tailings. Pb pollution in the study area can be linked to two primary anthropogenic sources: mining activities, which release accessory elements like Pb and Zn into the soil, and vehicle emissions, which contribute to atmospheric Pb deposition [55]. Honey samples from Dembi Dollo Market, Anafilo, and Gawo Kebe exhibited elevated levels of toxic metals, with Cd and Pb concentrations surpassing FAO/WHO and Codex Alimentarius regulatory limits [52]. This contamination, which compromises honey quality despite its nutritional value, primarily results from environmental factors. Heavy metals enter the apian product through bees foraging contaminated nectar and pollen from polluted flora. The main anthropogenic sources are mining operations, vehicular emissions, and the application of agricultural/industrial chemicals. Notably, pesticide use introduces metallic elements into ecosystems, which subsequently bioaccumulate in honey [53]. In general, honey contamination in the study area stems from both environmental exposure (air, water, pollen) and human activities. Additionally, factors such as poor beekeeping practices and the use of unsuitable containers that can leach heavy metals also contribute. Building on these findings, the study recommends that regulatory bodies establish continuous environmental monitoring and enforce stricter controls on agricultural and industrial emissions near apiaries. Furthermore, beekeepers should be encouraged to transfer hives away from polluted hotspots and adopt safe practices, including the use of food-grade storage containers.

### 3.2. Comparison of Heavy Metal Contamination in Honey from Three Study Areas

**Figure 2:**
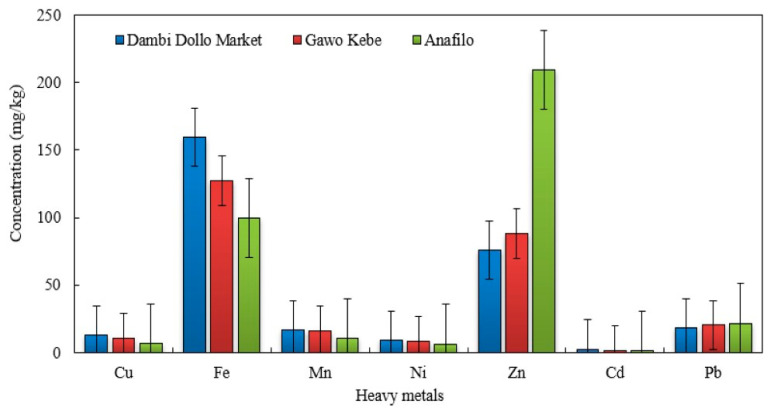
Comparison of heavy metal concentration in honey samples collected from the three study areas.

Heavy metal concentrations in honey varied across the three sites (Figure 2). Dambi Dollo had significantly higher levels of Cu, Fe, Mn, Ni, and Cd than Gawo Kebe and Anafilo (*p* < 0.05), suggesting a greater anthropogenic influence. In contrast, Zn was highest in Anafilo, while Pb increased from Dambi Dollo to Anafilo. Specifically, Cu, Fe, Mn, Ni, and Cd ranked Dambi Dollo > Gawo Kebe > Anafilo; Zn, Anafilo > Gawo Kebe > Dambi Dollo; and Pb, Anafilo > Gawo Kebe > Dambi Dollo. These trends reflect site contamination and variation in honey’s bioaccumulation.

### 3.3. Comparison of Heavy Metal Levels with Major Countries and Regions

A comparative analysis demonstrates that honey from the Kellem Wollega Zone contains substantially higher concentrations of heavy metals than those reported in honey from diverse international locations (Table 5). The levels of Cu and Fe in all the samples from study were lower than those of Turkish honey (Cu: 0.22–198.36 mg/kg, Fe: 3.51–1278.78 mg/kg) [53], but the Cu concentrations were higher than those of Iran (0.028–2.873 mg/kg) [62], Romania (0.064–0.549 mg/kg) [54], Armenia (0.09–1.86 mg/kg) [55] and Serbia (0.09–0.92 mg/kg) [67]. The concentration of Fe in all the samples from the study was higher than that in the Oromia Special Zone, Ethiopia (4.87–11.79 mg/kg) [66] and Serbia (0.77–3.94 mg/kg) [67]. The concentration of Mn in all the samples from the study was a lower value than the concentration of those obtained in Turkish honey (0.096–29.496 mg/kg) [53], but a higher concentration than those found in Romania (0.495–8.652 mg/kg) [54] and Ethiopia (ND–7.29 mg/kg) [68]. The concentrations of Cd in this study exceeded those reported in Iran (0.0014–0.126 mg/kg) [62], Romania (0.001–0.012 mg/kg) [54], and Australian honey (0.0025–0.053 mg/kg) [56]. The levels of Pb were at higher concentrations than those found in Armenia (0.002–0.045 mg/kg) [55], Romania (0.020–0.142 mg/kg) [54], and Australian honey (0.0025–0.69 mg/kg) [56]. The levels of Ni in all the samples from the study were higher than those found in Armenia (0.240–0.849 mg/kg) [55], Romania (0.101–0.570 mg/kg) [54], and Australian honey (0.005–1.4 mg/kg) [56] (Table 5). This pronounced elevation indicates significant localized environmental contamination, likely stemming from anthropogenic sources such as industrial emissions, agricultural chemicals, and traffic pollution, and identifies the areas as potential hotspots of heavy metal pollution.

### 3.4. Potential Health Risks Assessment 

The potential health risks from heavy metal intake through honey consumption were evaluated by calculating the average daily dose (ADD), target hazard quotient (THQ), hazard index (HI), and total carcinogenic risk (TCR).

#### 3.4.1. Non-Carcinogenic Health Risk

**Figure 3:**
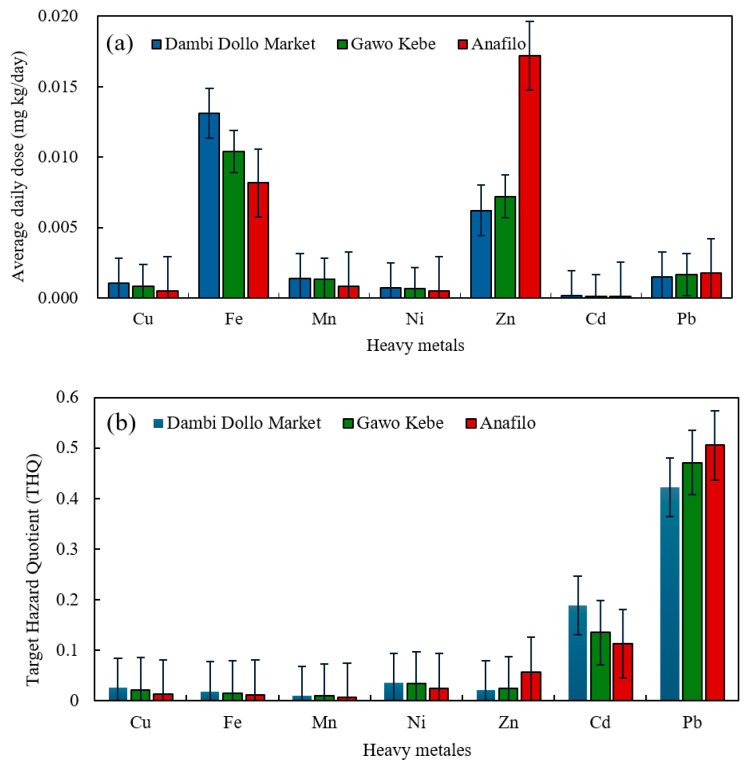
Comparison of (**a**) Average Daily Dose (ADD) and (**b**) Target Hazard Quotient (THQ) for heavy metals resulting from adult honey consumption across the three study areas.

The calculated Average Daily Dose (ADD) of heavy metals from honey consumption in Dambi Dollo ranged from $1.89\times 10^{-4}\;to\;1.31\times 10^{-2}$ mg/kg/day. Fe was the predominant metal across the study area, followed by Zn and Pb. Specifically, in Gawo Kebe, ADD values ranged from $1.35\;\times \;10^{-4}$ to $1.04\;\times \;10^{-2}$ mg/kg/day, maintaining the consistent order of Fe > Zn > Pb > Mn > Cu > Ni > Cd (Table 6 and Figure 3). This sequence consisted of Dambi Dollo. However, in Anafilo, ADD values ranged from $1.13\;\times \;10^{-4}$ to $1.72\;\times \;10^{-2}$ mg/kg/day, and Zn was more prevalent than Fe. Thus, the order was Zn > Fe > Pb > Mn > Ni > Cd. The Cd consistently exhibited the lowest average daily dose (ADD) across all sites, suggesting minimal intake from honey consumption. Additionally, the Average Daily Dose (ADD) values (mg/kg/day) for all investigated trace metals remained below the maximum tolerable daily intake (MTDI) thresholds for Cu (3.0), Mn (5.0), Cd (0.07), and Zn (60.0) [38,69,70], indicating that individual exposure to these metals through honey consumption does not pose a significant chronic toxic risk.

However, ADD values of Zn, Ni, and Cd were higher than those previously reported from Iran [71], suggesting relatively higher exposure in the present study areas. The target hazard quotients (THQs) for heavy metals in honey (Table 6, Figure 3) varied by site: Dambi Dollo, Gawo Kebe, and Anafilo. THQ values ranged from $1.29\;\times \;10^{-2}$ to $2.63\;\times \;10^{-2}$ for Cu; $1.17\;\times \;10^{-2}$ to $1.87\;\times 10^{-2}$ for Fe; $6.00\;\times \;10^{-3}$ to $9.87\;\times \;10^{-2}$ for Mn; $2.50\;\times \;10^{-2}$ to $3.63\;\times \;10^{-2}$ for Ni; $2.07\;\times \;10^{-2}$ to $5.73\times 10^{-2}$ for Zn; $1.31\;\times \;10^{-1}$ to $1.89\;\times \;10^{-1}$ for Cd, and $4.22\;\times \;10^{-1}$ to $5.06\;\times \;10^{-1}$ for Pb. In Dambi Dollo, the order was Pb > Cd > Mn > Ni > Cu > Zn > Fe. For Gawo Kebe, it was Pb > Cd > Mn > Ni > Cu > Fe. In Anafilo, Pb > Cd > Zn > Mn > Ni > Cu > Fe. Therefore, the highest calculated Target Hazard Quotient (THQ) for lead (Pb) was obtained in the Anafilo area. All THQ values calculated were well below 1, indicating no significant non-carcinogenic risk from individual metals in honey. The cumulative hazard index (HI) was highest in Dambi Dollo ($8.10\;\times \;10^{-1}$), followed by Gawo Kebe ($7.94\;\times 10^{-1}$) and Anafilo ($7.86\;\times \;10^{-1}$). As HI values were below 1, cumulative heavy metal exposure through honey does not pose a significant chronic toxic risk in these areas. These findings align with those from Zhejiang, China [72],and Southwest Ethiopia [26], where THQ and HI remained below 1. The results show that metal accumulation differs among sites, mainly due to local environmental conditions and anthropogenic factors. Prolonged exposure to these metals through honey does not pose a significant health risk. Using a hierarchical reference dose (RfD) ensures the risk assessment is reliable and comparable. This method provides a solid basis for evaluating human health risks.

#### 3.4.2. Carcinogenic Health Risk

The total cancer risk (TCR) for the carcinogenic metals Cd, Ni, and Pb ranged across the sampling sites. The calculated TCR values ranged from $4.29\;\times \;10^{-5}$ to $7.18\;\times \;10^{-5}$ for Cd; $8.50\;\times \;10^{-4}$ to $1.23\;\times \;10^{-3}$ for Ni, and $1.27\;\times \;10^{-5}$ to $1.50\;\times \;10^{-5}$ for Pb across the three study areas. The order of TCR contribution by site for each metal was: Cd: Dambi Dollo > Gawo Kebe > Anafilo, Ni: Dambi Dollo > Gawo Kebe > Anafilo, and Pb: Anafilo > Gawo Kebe > Dambi Dollo. Notably, Ni presents the highest carcinogenic risk across all three sites, with Cd as the secondary carcinogenic risk. In contrast, Pb represents a major noncarcinogenic hazard at all sites. Although the individual cancer risk values exceeded the established health guidance benchmark of $1\;\times \;10^{-1}$, they remained within the broader acceptable regulatory range of $1.0\;\times \;10^{-6}\;$to $1.0\;\times \;10^{-3}$ [73]. The cumulative carcinogenic risk (∑TCR) was determined to be $1.32\;\times \;10^{-3}$ for Dambi Dollo, $1.21\;\times \;10^{-3}$ for Gawo Kebe, and $9.08\;\times \;10^{-4}$ for Anafilo. According to the New York State Department of Health Center for Environmental Health, TCR categories are defined as follows: TCR < $1.06\;\times \;10^{-6}$ is low; $1.0\times \;10^{-4}$ to $1.0\;\times \;10^{-3}$ is moderate; $1.0\;\times \;10^{-3}$ to $1.0\;\times \;10^{-1}$ is high; and >$1.0\;\times \;10^{-1}$ is very high [73,74]. The carcinogenic risk is an estimate of the probability that a person will develop cancer over their estimated 55-year lifespan. Risk values greater than $1.0\;\times \;10^{-4}$ are considered unacceptable, risks below $1.0\;\times \;10^{-6}$ do not result in any adverse health effects, and risks between $1.0\times 10^{-4}$ and $1.0\;\times \;10^{-6}$ are generally considered in the safe range [74,75]. The elevated carcinogenic risk, particularly at Dambi Dollo, is associated with increased anthropogenic pressure and metal exposure in that area. However, the Pb value falls within the range reported [35,76]. The TCR values reported by [37] in Nigeria ($3.78\;\times \;10^{-2}$ to $6.05\;\times \;10^{-2}$) were much higher than those in the present study. However, our results were similar to levels reported by [19]. Therefore, the findings indicate a perceptible health risk to the local residents. The calculated cancer risk exceeds the regulatory benchmark of $1.0\;\times \;10^{-4}$. This means that chronic honey consumption from the study area could increase consumers’ lifetime risk of developing cancer beyond the accepted level. These findings impose direct investigation into potential contamination sources, such as local industrial emissions and improper electronic waste disposal.

## 4. Conclusions

This study shows that honey is a sensitive bio-indicator of environmental contamination. We analyzed honey samples from Dambi Dollo, Gawo Kebe, and Anafilo using ICP-OES after wet acid digestion with HNO_3_ and HClO_4_ (2:1.5 *v*/*v*). This method demonstrated high efficiency and accuracy, with recovery rates ranging from 89.09% to 114.82% and %RSD values between 0.12% and 1.35%, indicating accuracy and consistency. The analysis of heavy metals was in the order of: Zn > Fe > Pb > Mn > Cu > Ni > Cd. Metal concentrations varied significantly (*p* < 0.05) between sites, reflecting local human activities. Mining, especially in Anafilo, appears to be a major source of contamination. Pb and Cd levels exceeded established safety thresholds for environmental and human health. The health risk assessment found that the Average Daily Dose (ADD), the Target Hazard Quotient (THQ) for each metal, and the total Hazard Index (HI) were all below 1.0. There is no immediate non-carcinogenic health risk from exposure to these metals in this study. Target Cancer Risk (TCR) values for Ni, Cd, and Pb indicate a serious long-term carcinogenic risk. The cumulative TCR (∑TCR) for each study site, in the order Dambi Dollo > Gawo Kebe > Anafilo, exceeded the acceptable limit of $1\;\times \;10^{-4}$. This indicates a potential lifetime cancer risk from combined exposure, with Ni being the main contributor to total carcinogenic risk. We recommend routine monitoring, source identification, honey pretreatment before sale, public awareness, and the use of honeybees for pollution studies. Future research should assess health risks for vulnerable groups, including children, through local dietary surveys. Bioaccessibility tests will improve exposure estimates.

## Figures and Tables

**Table 1:** Optimal operating parameters for ICP-OES to identify specific heavy metals.

Parameters	Values	
RF power	1500 W	
Plasma gas flow rate	10 L/min	
Auxiliary gas flow rate	0.3 L/min	
Nebulizer gas flow rate	0.7 L/min	
Peristaltic pump flow rate	1 mL/min	
Replicates	3	
Element wavelength (nm)	Cu, 327.393	Cd, 228.802
	Fe, 238.204	Zn, 206.200
	Mn, 257.610	Pb, 220.353
	Ni, 231.604	

**Table 2:** Calibration data, limits of detection and quantification for the target metals analyzed by ICP-OES.

Metals	Standard Solutions Conc.(mg/L)	R^2^	IDL (mg/L)	MDL (mg/L)	LOQ (mg/L)
Cu	0.05, 1.05, 2.05, 3.05, 4.05, 5.05	0.996	0.014	0.028	0.057
Fe	0.05, 1.05, 2.05, 3.05, 4.05, 5.05	0.998	2.144	2.655	7.157
Mn	0.05, 1.05, 2.05, 3.05, 4.05, 5.05	0.999	0.057	0.071	0.185
Ni	0.05, 1.05, 2.05, 3.05, 4.05, 5.05	0.997	0.028	0.043	0.114
Cd	0.05, 1.05, 2.05, 3.05, 4.05, 5.05	0.996	0.099	0.128	0.327
Zn	0.05, 1.05, 2.05, 3.05, 4.05, 5.05	0.995	1.122	1.377	3.735
Pb	0.05, 1.05, 2.05, 3.05, 4.05, 5.05	0.998	0.469	0.582	1.576

IDL: Instrument Detection Limit, MDL: Method Detection Limit, LOQ: Limit of Quantification.

**Table 3:** Method validation: Recovery of target metals from spiked honey samples (*n* = 3).

Metals	^a^ Conc. in Sample (mg/kg)	Amount Added (mg/kg)	^b^ Conc. In Spiked Sample (mg/kg)	Recovery (%)	^c^ RSD (%)
Cu	10.46 ± 0.01	10.00	20.58 ± 0.35	101.2	1.70
Fe	127.20 ± 0.39	160	287.69 ± 2.59	100.31	0.31
Mn	16.08 ± 0.12	24.00	39.76 ± 0.60	98.6	1.48
Ni	8.19 ± 0.01	290	289.89 ± 1.47	97.14	0.15
Cd	1.65 ± 0.01	200	186.18 ± 1.74	92.27	0.49
Zn	88.02 ± 0.52	150	221.65 ± 1.05	89.09	0.59
Pb	20.15 ± 0.27	700	823.87 ± 5.70	114.82	1.35

^a^ Values represent the mean ± standard deviation of three replicate measurements. ^b^ Spiked concentration values represent the mean ± standard deviation of three replicate measurements. ^c^ RSD = relative standard deviation in percentage.

**Table 4:** Concentration (mg/kg) of heavy metals (*mean ± SD, n* = 3).

Metals	Sampling Sites		
	Dambi Dollo	Gawo Kebe	Anafilo
Cu	12.79 ± 0.01 ^a^	10.46 ± 0.01 ^b^	6.36 ± 0.02 ^c^
Fe	159.67 ± 0.57 ^a^	127.20 ± 0.39 ^b^	99.58 ± 0.77 ^c^
Mn	16.73 ± 0.07 ^a^	16.08 ± 0.12 ^a^	10.23 ± 0.06 ^c^
Ni	8.85 ± 0.02 ^a^	8.19 ± 0.01 ^b^	6.05 ± 0.01 ^c^
Zn	75.71 ± 0.40 ^c^	88.02 ± 0.52 ^b^	209.54 ± 1.01 ^a^
Cd	2.30 ± 0.02 ^a^	1.65 ± 0.01 ^b^	1.38 ± 0.01 ^c^
Pb	18.00 ± 0.13 ^NS^	20.15 ± 0.27 ^NS^	21.42 ± 0.18 ^NS^

The different lowercase superscript letters (^a^, ^b^, ^c^) within a row indicate statistically significant differences between sites, the same letter indicates no significant difference (*p* < 0.05, Tukey’s HSD test), ^NS^: not significant (*p* > 0.05, one-way ANOVA). SD = Standard deviation, n = Number of replicate measurements.

**Table 5:** Comparative Analysis of Heavy Metal Concentrations in Honey Across Various Countries and Regions.

Country, Analytical Instrument, (Unit)	Concentration of Metals (mg/kg)							Reference
	Cu	Zn	Cd	Pb	Fe	Ni	Mn	
Iran	0.028–2.873	0.123–6.639	0.0014–0.126	0.117–1.63	#	0.065–1.094	#	[62]
Turkey	0.22–198.36	1.73–245.21	0.000–0.300	0.000–3.04	3.51–1278.78	#	0.096–29.496	[53]
Romania	0.064–0.549	0.742–8.012	0.001–0.012	0.020–0.142	11.355–54.785	0.101–0.570	0.495–8.652	[54]
Armenia	0.09–1.86	0.420–2.32	0.001–0.003	0.002–0.045	#	0.240–0.849	#	[55]
Serbia	0.09–0.92	0.37–8.02	0.001–0.01	0.004–0.026	0.77–3.94	#	#	[67]
Ethiopia	0.02–1.15	9.96–16.03	ND–0.017	ND–2.53	#	ND	ND–7.29	[68]
Ethiopia	0.22–1.22	1.41–6.94	0.04–0.70	0.37–0.90	4.87–11.79	0.26–0.60	#	[66]
Australian	0.05–4.8	0.16–120	0.0025–0.053	0.0025–0.69	0.2–99.0	0.005–1.4	0.38–38.0	[56]
Ethiopia	6.36–12.79	75.71–209.54	1.38–2.30	18.00–21.42	99.58–159.67	6.05–8.85	10.23–16.73	Present study

where ND = Not detected, # = Not reported.

**Table 6:** Health risk assessment: Average daily dose (ADD), target hazard quotient (THQ), hazard index (HI), and target cancer risks (TCRs) of heavy metal in honey consumption.

**(A) Non-Carcinogenic Health Risk**	**Metals**	**Sampling Sites**		
		**Dambi Dollo**	**Gawo Kebe**	**Anafilo**
Average daily dose (ADD, mg/kg/day)	Cu	1.05 × 10^−3^	8.59 × 10^−4^	5.18 × 10^−4^
	Fe	1.31 × 10^−2^	1.04 × 10^−2^	8.17 × 10^−3^
	Mn	1.37 × 10^−3^	1.32 × 10^−3^	8.39 × 10^−4^
	Ni	7.26 × 10^−4^	6.68 × 10^−4^	5.00 × 10^−4^
	Zn	6.22 × 10^−3^	7.22 × 10^−3^	1.72 × 10^−2^
	Cd	1.89 × 10^−4^	1.35 × 10^−4^	1.13 × 10^−4^
	Pb	1.49 × 10^−3^	1.65 × 10^−3^	1.77 × 10^−3^
Target hazard quotient (THQ)	Cu	2.63 × 10^−2^	2.15 × 10^−2^	1.29 × 10^−2^
	Fe	1.87 × 10^−2^	1.48 × 10^−2^	1.17 × 10^−2^
	Mn	9.78 × 10^−2^	9.43 × 10^−2^	6.00 × 10^−3^
	Ni	3.63 × 10^−2^	3.34 × 10^−2^	2.50 × 10^−2^
	Zn	2.07 × 10^−2^	2.40 × 10^−2^	5.73 × 10^−2^
	Cd	1.89 × 10^−1^	1.35 × 10^−1^	1.13 × 10^−1^
	Pb	4.22 × 10^−1^	4.71 × 10^−1^	5.06 × 10^−1^
Hazard Index (HI)		8.10 × 10^−1^	7.94 × 10^−1^	7.86 × 10^−1^
**(B) Carcinogenic health risk**	**Metals**	**Dambi Dollo**	**Gawo Kebe**	**Anafilo**
Target Cancer Risk (TCR)	Cd	7.18 × 10^−5^	5.13 × 10^−5^	4.29 × 10^−5^
	Ni	1.23 × 10^−3^	1.14 × 10^−3^	8.50 × 10^−4^
	Pb	1.27 × 10^−5^	1.40 × 10^−5^	1.50 × 10^−5^
Total Cancer Risk (∑TCR)		1.32 × 10^−3^	1.21 × 10^−3^	9.08 × 10^−4^

## Data Availability

All data generated or analyzed during this study are included in this article. Further enquiries can be directed at the corresponding author.
